# Measuring Beyond the Standard: Informal Measurement Systems as Cognitive Technologies

**DOI:** 10.1111/tops.12770

**Published:** 2024-11-10

**Authors:** Roope O. Kaaronen, Mikael A. Manninen, Jussi T. Eronen

**Affiliations:** ^1^ PAES Research Unit, Faculty of Biological and Environmental Sciences University of Helsinki; ^2^ BIOS Research Unit

**Keywords:** Measurement, Cognitive technology, Heuristics, Ethnometrology, Historical metrology, Cultural evolution, Rules of thumb

## Abstract

This paper explores the role of measurement as a cognitive technology across human history, emphasizing the coexistence of formal and informal measurement systems. While standardized systems dominate contemporary culture and are well documented across large‐scale societies of the past, this manuscript highlights the less explored domain of informal measurement practices that have been integral to daily life from the past to the present. Through the examination of body‐based measurement systems and proportional heuristics, we demonstrate how these informal strategies were not merely precursors to formal standards but essential adaptive tools for solving everyday problems. Often, these informal solutions come with practical advantages. This manuscript calls for a broader recognition of their significance in cultural and technological evolution.

## Abstract 2

This paper examines measurement as a cognitive technology, focusing on the coexistence of formal and informal systems. Through analysis of body‐based units and proportional heuristics, it reveals how informal measurement practices have been important tools in shaping culture and technologies from the past to the present.

## 1 Introduction

Throughout history and across diverse cultures, measurement has played an essential role as a cognitive technology, enabling humans to quantify, compare, analyze, and recombine their material world (Cooperrider & Gentner, 2019; Gyllenbok, 2018; Treese, 2018). Here, we understand cognitive technologies as culturally acquired knowledge systems that organize or manage information in ways that shape symbolic and material culture. The act of measuring is fundamental to forming our physical and social environments, proving indispensable for the evolution of material technologies and societal institutions.

Measurement can be considered a cognitive technology that scaffolds cultural niche construction: Not only do measurement units allow the direct quantification and thus manipulation of natural and cultural phenomena, but they also themselves inspire subsequent sets of analogies that can result in complex and interrelated systems of measure (Cooperrider & Gentner, 2019). Yet, despite their significance, the methods and practices of measurement in the daily lives of people in pre‐industrial societies remain relatively underexplored. Modern standardized measurement systems are only a few centuries old—how did humans measure things before their development or adoption?

When the concept of measurement is mentioned, many envision the systems that dominate today, such as the International System of Units or the metric and imperial systems. In the present age, standardization permeates every aspect of our existence, from the precise calibration of temperatures to the uniformity of text on this page. This widespread formalization and its implications have not gone unnoticed by scholars, who have thoroughly investigated historical systems of weights and measures (Gyllenbok, 2018; Treese, 2018), while also critically reflecting on the broader implications of living in a world where almost everything is meticulously defined and regulated (Busch, 2011; Scott, 1998; Weber, 1930).

Yet, on the timescale of human evolution, formal systems of measurement are a relatively recent innovation. The origins of formal measurement systems date back approximately 4000 to 5000 years, and their widescale adoption followed much later (Gyllenbok, 2018; Kaaronen, Manninen, & Eronen, 2023a; Kenoyer, 2010). Typically, such early cases of standard measures are documented in top‐down administrative contexts, such as taxation or large‐scale construction projects in early imperial societies. In other words, early standards were not typically^1^ in wide use by laypeople, and even in more recent history, the adoption of standards has faced widespread opposition (Crosby, 1997; Rooney, 2021; Vincent, 2022).

Historical analyses have traditionally focused on formal, standardized measurement systems, which is understandable given the tangible evidence these systems leave behind, such as stone weights (Gear & Gear, 2002), metal fragments (Ialongo & Lago, 2021), and copper rods (Duran & Aydar, 2012). These artifacts serve as the main evidence for ancient measurement practices and rightly highlight the significance of standardization in cultural and technological evolution. But while such standards undoubtedly facilitated governance, commerce, and technological change, this focus introduces a survivorship bias by overlooking the many informal, non‐standardized measurement systems that were essential to daily life throughout (pre)history. These informal systems, being more ephemeral cognitive technologies, often left little or no physical trace, and thus are much more elusive targets for scholarly inquiry.

Recent research indicates that even after standardized measurement systems became widespread, they were not necessarily applied in daily activities (Riggsby, 2019). Additionally, we have shown that cultures worldwide have employed other kinds of informal yet resourceful measurement strategies to solve everyday problems even when standards were available (Kaaronen et al., 2023a; Kaaronen, Manninen, & Eronen, 2023a, 2023b). Below, we first discuss this “double life” of measurement, describing how formal and informal measurement systems have coexisted. Then, based on our previous work, we present two reviews of informal measurement—body‐based measurement systems and heuristics—discussing how and why these cognitive technologies were used up until the recent past to solve everyday problems. To conclude, we argue that to fully respect the role of measurement as a cognitive technology in human cultural and technological evolution, and to understand how humans have measured things before and alongside standards, we must account for the existence and use of both formal and informal measurement systems.

## 2 The double life of measurement

Measurement standards first emerged within early large‐scale societies or empires (Gyllenbok, 2018; Treese, 2018; Kaaronen et al., 2023a, Supplementary Table 1). Typically, these cognitive technologies were developed to enable the practice of statecraft. Much like early writing systems (Steinkeller, 2004), early measurement standards were often employed to render a complex social organization more legible (Scott, 1998): to enable commensurability in administration, commerce, and construction projects. Such is the case in Old Kingdom Egypt, for example, where the royal cubit—perhaps the oldest known measurement standard (ca. 4700 BP)—was used in the design of the early pyramids (Stone, 2014, p. 4) and in taxation, where cubits were used to define the volumetric units for grain measurement (Hirsch, 2013, p. 120). Other early standard Egyptian means of measurement were employed in the measurement of gold (Petruso, 1981) and surveying of land (Ezzamel, 2002; Gyllenbok, 2018, Chapter 19). Such early applications of measurement standards generally did not align with the daily experiences of ordinary people, who would be unlikely to engage in major construction projects, land surveys, or the weighing of precious metals. Much like early writing technologies (Steinkeller, 2004) and numeral systems (Chrisomalis, 2020, Chapter 2), standards often did not serve the direct needs of the layperson.

This raises an important question: To what extent did standard units of measurement influence the daily lives of people in early societies with standardized systems? And if these standards were not aligned with the everyday practices of ordinary people, what alternatives might they have used to address their needs? Solving the puzzle of non‐standard measurement practices is challenging, primarily because informal units of measurement are less likely to be documented or preserved, making them difficult to detect in historical or archaeological records. Regardless, in the past years, some noteworthy evidence has emerged that we discuss in the rest of this manuscript.

Recent analyses of ancient lay measurement systems suggest that the impact of standardization on daily life may have been less significant than previously believed. In a study on Roman measurement systems, Riggsby (2019, Chapter 3) argues that despite efforts toward standardizing weights and measures in ancient Rome, these initiatives often failed to achieve their intended goals. This suggests that even in one of the more systematically organized societies of its era, the day‐to‐day lives of ordinary people were not profoundly influenced by standardization efforts.

The lack of standardization appears in at least two forms. First, even though standards were established, in practice, they were interpreted or used more colloquially. Take, for example, the Roman measure *passus* (pace), which despite being a standardized linear measure was used as more of an analogy (Riggsby, 2019, p. 91). Thus, when Roman writers refer to something being “a hundred paces” away, this should typically not be interpreted literally. Instead, such estimates portray an approximation, a rough imaginary traversal of space (Riggsby, 2019, p. 91). Similar is the case of volumetric units such as cups and spoons (*acetabulum* and *cyathus*): Although abstract standard definitions for these measures existed, people would generally prefer the use of an actual cup or spoon, not utilizing the strict definitions or full scope of sub‐units that these standards would afford (Riggsby, 2019, pp. 89–93).

Second, even where standards were established, they often were unreliable and varied considerably. This is emblematic of measurement systems throughout history, up until the recent past. As recently as the year 1800, an astounding 112 varying standards for Elles (cubits) and 123 Eimers (buckets) have been documented in German regions alone (Gyllenbok, 2018, p. 1140). Such variation would notably diminish the usefulness of early standards. In Rome, too, various local standards coexisted, with little attempt (or, at least, success) at large‐scale harmonization (Riggsby, 2019). The standard measures of length and weight in Rome have been shown to have had considerable error rates, which may have rendered official standards less useful for those practical crafts, such as carpentry or masonry, that demanded actual precision (Riggsby, 2019, pp. 100–101). Those domains of life closer to statecraft, such as state‐led land surveys, may have seen stricter use of standards (Riggsby, 2019, p. 114), but for practical crafts, a standard may ironically have been too inaccurate to reliably utilize.

We argue that measurement systems have lived parallel lives, with informal systems persisting alongside formal ones (Kaaronen et al., 2023a). This “double life” of measurement has seen formal standards defined by administrative organs, all the while the daily lives of laypeople have been characterized by more informal solutions. Consequentially, if we wish to gain a more thorough perspective of measurement as a cognitive technology, we must shift our attention beyond standards, also analyzing those informal means of measurement so often used by local peoples to solve everyday problems.

Next, we turn our attention to some ways in which measurement is known to have been used in frameworks of practical and traditional knowledge. Our focus here is exclusively on two common forms of practical measurement that we have previously reviewed: body‐based units (Kaaronen et al., 2023a) and heuristics that make use of proportions or ratios (Kaaronen et al., 2023b). We illustrate how these cognitive technologies afforded various kinds of resourceful problem‐solving strategies that often addressed the direct needs of local lifeways—without reference to standards.

## 3 Measuring with the body

A common feature of early measurement standards is their derivation from units based on the human body. This highlights the intrinsic connection between human everyday embodied experience and the quantification of the physical world (Lakoff & Núñez, 2000). Much like how our 10 fingers and toes have inspired a variety of numeral and counting systems (Bender & Beller, 2012; Heine, 1997), our bodily proportions have fundamentally shaped how we measure our surroundings. We have already discussed two body‐derived units, the Egyptian cubit (*mahe*), which was inspired by the length of the human forearm, and the Roman *passus*, which derived from the human pace. However, this derivation of early standards from the human body is a recurrent trend across societies worldwide.

For example, Harappan measurement systems standardized units such as the finger breadth (*aṅgula*; Balasubramaniam, 2009; Shrivastava, 2017), hand span, cubit, fathom (span of outstretched arms), and pace (Kenoyer, 2010). Various ancient Mesopotamian measurement units (e.g., in Akkadian, Sumerian, and Assyrian systems) were abstracted from body proportions such as the foot, finger, cubit, and step (Treese, 2018). Chinese measurement systems have used standards such as the thumb‐width (*cun*) and foot (*chi;* Keightley, 1995). Greek and Roman systems involved measures such as the standardized foot, finger width, pace, and cubit (Gyllenbok, 2018; Pollio, 1914; Riggsby, 2021). Similar standardized body‐derived units are further documented in Aztec (Clark, 2010) and Maya (Gyllenbok, 2018; O'Brien & Christiansen, 1986) measurement systems. This evidence, along with linguistic data (e.g., Wichmann, 1927), suggests that before these standardized systems were formalized, people likely relied on their bodies for direct measurements.

However, owing to their informality, we know relatively little of the prehistoric use of true body‐based measurement systems. Here, we use the concept of “body‐based units of measure” to refer exclusively to those measurement systems that are directly measured using one's body, thus distinguishing them from the *body‐derived* standards described above. According to ethnographic reviews (Cooperrider & Gentner, 2019; Kaaronen et al., 2023a), body‐based units of measure appear ubiquitous across contemporary societies and seem to have been some of the most common informal solutions to measuring. We have documented such units across 186 cultures worldwide, covering over half of the cultures in the Standard Cross‐Cultural Sample (Kaaronen et al., 2023a). Some of the most common cross‐culturally occurring body‐based units are defined in Table 1.

**Table 1:** The 20 most common body‐based units of measure across contemporary ethnographic records, adapted from Kaaronen et al. (2023a)

Unit of Measure	Description and Variations
*Fathom*	The distance between fingertips of outstretched arms. The fathom can be adjusted by using sections of the arms or hands as reference points, for example, by measuring the fathom with closed fists, or the fathom to the opposite elbow crease. Also known as the arm span
*Hand span*	On a hand with all fingers extended, the distance from the tip of the thumb to the tip of another finger (most commonly, the index finger or little finger)
*Cubit*	Also known as the ell. Measured from the tip of the elbow to the tip of an extended finger (commonly, middle finger) on the same arm. Variations include taking the measure from the elbow to another reference point, such as the tip of a closed fist or the wrist crease or measuring from the elbow crease to the fingertips
*Arm length*	The length of the arm, most commonly from the tip of an outstretched finger on an outstretched arm to one of the following reference points: the armpit, shoulder, or middle of the chest (the latter equals a half‐fathom)
*Finger width*	The width of a finger, or the width of several fingers when combined. Sometimes also measured using just the fingernail
*Hand width*	The width of the palm with all fingers closed together, also known more simply as the “palm.” Variations include the width of the closed fist and the circumference of the palm
*Pace*	A pace, step, or stride, typically measured while walking
*Finger length*	The length of one of the four fingers. Variations include taking the length to a specific finger joint or crease
*Height*	A measure based on a person's height, typically from the ground to the tip of the head. Many variations exist, such as taking the measure up to the tip of vertically extended arms, or to any other anatomical point of reference (e.g., the navel, eyes, or forehead)
*Foot*	The inner or outer length of one's foot, typically from the end of the heel to the tip of the big toe or index toe
*Handful*	A volumetric measure, using a cupped hand (handful) or two cupped hands (double handful)
*Thumb width*	The width of the thumb (or thumbnail)
*Fistmele*	Using a gesture similar to “thumbs up,” measures the height or width of the fist to the tip of an extended thumb
*Thumb length*	The length of the thumb. Variations can take the measure to the crease of a specific thumb joint
*Hand length*	The length of one's hand, typically measured from the wrist crease to the tip of the extended middle finger
*Arm thickness*	The thickness (girth) of the arm
*Armful*	A volumetric measure, based on the amount of material a person can carry in both arms
*Pinch*	A smaller volumetric measure taken by pinching the thumb against the tip of a finger (e.g., a “pinch of salt”)
*Leg length*	The length of a leg, typically measured from the sole of the foot to a reference point such as the hip, groin, or knee
*Ring*	A measure of girth or circumference by using a gesture similar to the “OK” sign, by pinching the tip of one's finger to the tip of the thumb

Our review shows that body‐based units of measure have persisted for centuries, and in some cases, millennia, even after the advent of standardized measurement systems (Kaaronen et al., 2023a). This highlights that not only is it likely that body‐based measurements have pre‐dated standard systems but also coexisted with them, playing an important role in how humans understand and conceptualize their environment up until contemporary times. Despite their informality, body‐based units of measure have enabled innovative technological applications, often with practical advantages which we discuss below.

### 3.1 Practical aspects of body‐based measurement

The contemporary perspective often frames measurement as a problem of representation—the quantitative expression of an entity using (abstract and standard) units (Gyllenbok, 2018). While this is agreeable from a metrological standpoint (assuming we seek precision), throughout much of history, the objective of measurement has also taken a more pragmatic nature: aiming for efficient problem‐solving with relative accuracy for a specific task (Fitchen, 1989; Kaaronen et al., 2023a, 2023b; Turnbull, 1993). Contemporary cognitive scientists would describe this as “satisficing,” seeking satisfactory yet sufficient strategies to solve a specific problem (Simon, 1956). Viewed through the lens of problem‐solving, many informal measurement solutions appear in a very different light.

First, consider the rather self‐evident benefit of a body‐based unit: Their length is relatively stable over time (at least after adolescence), and we always carry them with us. This renders body‐based units especially useful when other measurement technologies are not at hand (Kaaronen et al., 2023a). For a mobile population, such as hunter‐gatherer or pastoralist societies, such portability would be a practical boon since measurement could be conducted without reference to external, and often cumbersome, technologies. Further, the hand span or cubit, for example, is conveniently proximate to the visual system, affording the easy juxtaposition of these units with the measured target. Accordingly, when engaging in a manual task, such as sowing seeds, knotting a net, or measuring the distance between posts, these measurement units are conveniently available.

Yet the practicality of body‐based units extends well beyond their mere availability; their usage is often in synergy with the physical tasks they are employed in (Kaaronen et al., 2023a). This is most evident in the measurement of flexible items like ropes or fishing nets. Measuring such items is no trivial task since they are typically very long and, when measured, would require multiple extensions and generally cumbersome operations. Taking the measure of a rope with a yardstick or measuring tape can be inconvenient. Accordingly, especially when measuring a rope or net in a situation that requires quick reactions (e.g., when seafaring), other solutions would have to be invented—and the use of the fathom is a common response. Cultures around the world have been documented measuring ropes or nets with fathoms, taking their length by sequentially marking off and counting each full stretch of their arms, seamlessly moving segments of the rope from one hand to the other (Buck, 1957, p. 296; Densmore, 1929, p. 154; Firth, 1939, p. 100; Itkonen, 1984, p. 933; Kaaronen et al., 2023a, pp. 951–952; Sather, 1997, p. 117). In fact, the influence of such practices is still observable today: A standardized fathom is used as a maritime unit of measure for sounding sea depth in the imperial system of units.

### 3.2 Ergonomics

Ergonomics is commonly defined as a discipline born out of the modern, post‐industrial era. It is typically introduced in textbooks as emerging in response to the Taylorist principles of industrial productivity, and especially to practical design challenges faced in the Second World War (Meister, 2018). While it is true that new ergonomic designs had to be implemented following industrial mass production, the concept of designing tools and equipment to suit human needs and fit the “human scale” is in fact far from a modern invention. Throughout history and across cultural traditions, people have encountered repetitive tasks and physical strain (Bridges, 1992), problems that necessitated solutions that would minimize discomfort and increase efficiency. There has been a longstanding demand for tools that are functionally designed for human use, enabling effective problem‐solving and task performance. The question is then: what kinds of measurement principles have been used in designing ergonomic equipment? One prevalent approach to designing personalized tools has been the utilization of the human body itself as a frame of reference, ensuring that the design of tools and spaces was directly aligned with the user's physical dimensions (Kaaronen et al., 2023a).

Anthropometric design principles have taken many forms (Table 1) and, in the ethnographic literature, are almost always conducted without reference to formal measurement standards (Kaaronen, 2023). Our review documents body‐based anthropometric design principles in a variety of societies, typically used in the measurement of clothes, bows, spears, skis, agricultural tools, and various other essential equipment (Kaaronen et al., 2023a). We focus here on two such examples, both emblematic case studies of ergonomic design principles in traditional or Indigenous knowledge systems: the kayak and the bow.

The kayak (from Kalaallisut: *qajaq*) is a class of watercraft technologies originally found across circumpolar societies. Although kayaks vary in shape and size accordingly with their variable functions (Kankaanpää, 1989), they are in most cases traditionally designed with anthropometric principles (Arima, 1994; Heath & Arima, 2004; Robert‐Lamblin, 1980). An important aspect of kayak design involves a trade‐off between maneuverability and stability. Kayaks are typically designed to be nimble, allowing for quick and precise movement in varying water conditions and hunting tasks. However, this increased maneuverability can reduce stability, making kayaks more prone to tipping. To counter this, effective control through the paddler's body movements is required, which necessitates an ergonomically centered design approach. By using body‐based measurements, kayaks are crafted to respond to the paddler's movements, ensuring both control and adaptability, regardless of individual physical characteristics.

For reasons such as these, for example, a Yup'ik kayak is traditionally designed with a couple dozen units based directly on its user's body (Fienup‐Riordan, 2007, pp. 88–89). These measures define the lengths and widths of tens of separate parts that make up the dimensions of the kayak's wooden frame. Commonly used units include the *yagneq* (outstretched arm to tips of index fingers), *tekneq* (span from tip of thumb to index finger), *ikuyagneq* (cubit; from elbow to tip of index finger), *taluyarneq* (elbow of one arm to the tip of the index finger on the other extended arm), and *patneq* (width of the flat hand). The dimensions of the kayak are almost entirely based on the body (for a full schema, see Fienup‐Riordan, 2007, pp. 88–89).

Similar principles were used for the design of the paddle. Today, perhaps the most famous (and in increasingly widespread use) is the Greenland‐style double‐bladed paddle, which too was designed with human‐scale design (Heath & Arima, 2004). The paddle is typically the length of its user's fathom plus cubit, the girth of its loom is determined by pinching one's index finger to the thumb, and at width of the widest point of the blade is determined by making a C‐shape with the thumb and forefinger, ensuring that the blade is the maximum width one can comfortably grip. The latter results in a relatively narrow blade but ensures that the blade can be firmly gripped from both ends. This facilitates leveraging the full length of the paddle, easing wide strokes and the rolling of a capsized kayak. The implication is then that a kayak forms an anthropometric technological system: the pilot's dimensions determine not only the dimensions of the kayak parts but also the dimensions of the kayak paddle, which all together form a coherently measured whole.

Bows, much like the kayak, are often designed with anthropometrics in mind. This applies to both modern and traditional bows. Anthropometric measurements are used especially when determining the length of the bow, its draw length, string tension, and grip. Together, these ensure good energy transfer, control, durability, and shooting accuracy. Different types of bows, such as longbows, shorter hunting bows, and recurve bows, are designed with specific purposes in mind, leading to varying design requirements. For instance, longbows are usually taller with a longer draw length, making them ideal for distance and precision shooting, while shorter bows—such as those used for horseback archery—are more compact and maneuverable, allowing for quick, agile shots in confined spaces. This diversity in form and function means that no universal design standards exist. Like the kayak, each bow type must be tailored not only to the user's body but also to its intended application. Nevertheless, a common thread across many bow traditions is the principle of designing in accordance with the user's body dimensions.

Anthropometric bow measurement is especially well‐documented in North American Indigenous archery traditions. Altogether, ethnographic evidence is very explicit about the notion that body‐based units were favored for efficiency and ergonomics. The Yahi measured a bow's length from the right hip joint to the left fingertips, with the person standing upright and extending their left arm in a straight line from the shoulder (Kroeber, 1961, p. 190; Pope, 1920, p. 187). The width of the hand grip depended on the bow's intended power, using four fingers for a powerful bow and three for a lighter hunting bow. The arrow was measured by placing its butt against one's sternal notch and extending the left hand along the shaft in a near shooting position, then cutting it off at the tip of the left forefinger (Pope, 1918). The Piipaash would create bows extending from the ground to the maker's forehead (Spier, 1933, p. 132). Similarly, various Apache and Pueblo groups tailored bow lengths by measuring from the ground to anatomical reference points such as the lower ribs, armpit, upper chest, base of sternum, chest, or waist with variations depending on the bow's intended use (Gifford, 1940, p. 119). Ojibwe bow lengths corresponded to the stature of the owner, typically measured from the shoulder to the middle finger of the opposite hand (Densmore, 1929, p. 146). The Blackfoot people measured a bow's length with a half‐fathom, from the tip of the middle finger to the center of the chest (Levinson, 1995, p. 144).

Anthropometric bow‐design principles were not unique to North America, however. Historical records inform us that in 15th‐century England, King Edward IV mandated every male aged 16 to 60 to construct a longbow matching their own height, with a fistmele (a fist's width with the thumb extended) between the string and bow (Hansard, 1841). Similarly, 11th‐century Anglo‐Saxon bows depicted in the Bayeux Tapestry have been suggested to approximately match their users’ heights (Rees, 2016 ). In Japan, the asymmetrical Yumi (弓) bows (which also come in various sizes) have been determined at least partly using the user's height as a reference (Hideharu Onuma, 1993, pp. 42–43). In Arab archery traditions (Nabih & Elmer, 1945, pp. 59, 63), the space between the string and the grip of a braced bow has been measured using the span or half‐span of the bow's user (the span, *shibr*, was measured from the tip of the thumb to little finger; the half span, *fitr*, measured from thumb to index finger). The size of the grip should be proportional to the archer's hand, to ensure ease and comfort. The length of an arrow, depending on the source, was measured using combinations of body‐based metrics, including variations of the archer's cubit, forearm, leg or foot length, arm length (from armpit to the tip of the middle finger), or a specific number of the archer's fists (Nabih & Elmer, 1945, p. 63).

Examples such as these illustrate how non‐standard measurement solutions can be used to efficiently solve problems, especially when ergonomic design is required. Undoubtedly, many such anthropometric measurement systems have been lost to time—they leave little material trace, although their histories may yet be traced from traditional knowledge, ethnographies, and perhaps yet‐unanalyzed dimensions of archaeological artifacts.

That is not to say that the variability of body‐based units of measure is without its problems, most notably incommensurability and lack of precision. Certainly, early proponents of industrial standardization were explicitly intolerant against such “inefficient rule‐of‐thumb methods” (Taylor, 1911, p. 16). Accordingly, it has been suggested that standardized units of measure were developed particularly to ease social cooperation (Childe, 1951, p. 154). Although this must be true to an extent, it should be noted that universal standards are not completely necessary for collective problem‐solving. For instance, although the construction of a Yup'ik kayak typically begins by taking the body measures of the kayaks user, after the initial measures are taken, the kayak construction is a thoroughly communal and social activity (Fienup‐Riordan, 2007). Similarly, in a variety of construction traditions, the body‐based measures of the construction leader were taken first, establishing a local and temporary standard of sorts, after which cooperation could take place (Best, 1924; Kobayashi & Nguyen, 2014). Further, it should be noted that local peoples were often not oblivious of the limitations brought on by interpersonal variability of body‐based units. In fact, many cultures attempted to correct for such variability. The problem could be tackled by taking the measures of a person perceived as being average‐sized (Halayqa, 2015; Wichmann, 1927) or through the use of slightly different body‐based units by people of different sizes: A person with large hands, for instance, could use a different hand span measure to those with a more average sized hand (Felkin, 1886, p. 250; Hilger, 1957, p. 92; Itkonen, 1948, p. 930; Marshall, 1922, p. 51).

## 4 Ratios, heuristics, and rules of thumb

Next, we consider another informal measurement technique: the use of ratios and proportions in everyday tasks. Ratio‐based thinking is a versatile cognitive technology and a widely used approach for addressing measurement challenges across cultures. Most of us are familiar with this practice: take, for example, the common rule of thumb for cooking rice with two parts water for one part rice. With this method, almost any container can be used, as success depends less on precise units of measure and more on maintaining the correct proportion. In many daily situations, proportions can be scaled up or down without causing major issues. Such problem‐solving strategies may be described as making use of heuristics, or cognitive shortcuts, that allow efficient decision‐making in a specific context (Kaaronen et al., 2023b). Although cognitive scientists have studied heuristic problem‐solving among humans for decades (Simon, 1956, 1972; Todd & Gigerenzer, 2012), the everyday heuristics humans have used to solve problems across cultures and behavioral domains are still relatively poorly charted (but see Kaaronen et al., 2023b).

The use of ratios or proportions often bypasses the need for (but is not incompatible with) standard units. Our review describes a variety of cases where traditional knowledge frameworks have utilized proportions or ratio‐based heuristics (Kaaronen et al., 2023b). Consider, for instance, the longship, an essential technology for commerce, exploration, and warfare in the Viking Age. Analyses of longship construction have suggested that they were likely built by eye, without construction drawings, molds, or downscaled models, by illiterate artisans (Crumlin‐Pedersen, 1986, 2009). Therefore, it is unlikely that these ships were built using standard measures. Rather, longships were designed proportionally: one part was determined by the length or shape of another. For instance, the radii of the ship's stems may have been proportionally related to the length of the keel. These were relatively simple, often geometric, rules of thumb that served as useful “mental templates” (Dhoop & Olaberria, 2015), which were convenient to pass on from one craftsperson to another or from master to apprentice.

In a similar spirit to Viking longships, Marshallese outrigger canoes used various proportions in their design: the length of the canoe is equal to the length of the mast, the distance from the weather side of the hull to its outrigger float is half the canoe's length (Mason, 1947, p. 88). Simple proportions such as these could be learned by using mnemonics or uncomplicated tools such as story sticks or chalk lines, making them bite‐sized cognitive technologies apt for practical problem‐solving (Crumlin‐Pedersen, 1986, 2009). For approximately similar‐sized ships, these solutions satisficed and evidently could reproduce very successful vessels.

In the Roman world too, proportional values were often preferred to absolute ones (Riggsby, 2019). This is evident in Roman measurement practices from architecture (Vitruvius, 1960) to water allocation (Riggsby, 2019, Chapter 3). Even the Roman hour was proportional, with the Roman hour being one twelfth of the period from sunrise to sunset, making the hour anything from ca. 45 to 75 min depending on the season (Riggsby, 2019, p. 88). Although taxation could at times refer to standard units of measure, in practice, taxes were often calculated in ways that did not require standards at all, instead using established proportions to determine the amount of grain taxed (Riggsby, 2019, p. 108).

Similar proportional rules have been documented across the globe: They appear in the design of Saxon churches (James, 1982, pp. 33–34), Medieval cathedrals (Turnbull, 1993), Chinese roofs (Li et al., 2021), ancient Mediterranean ships (Olaberria, 2014) and have been utilized by blacksmiths (Rytkönen, 1931, pp. 143–146) and stonemasons (James, 1979) alike. Using such simple proportional or geometrical rules, even large‐scale construction projects could be managed as James (1979, 1982) has thoroughly documented in the construction of the Chartres cathedral—which despite being one of the most complex architectural achievements of its time, was not designed with reference to universal standard measures.

## 5 Conclusion

In research on historical metrology, the focus has mostly been on standard units of measure. Yet, from recent research, we may assume that in the past, such standards may have only played one part in the complex ways humans measure things. Throughout history, humans have also often relied on more informal solutions for everyday measurement. To gain a more comprehensive view of measurement as a cognitive technology, as well as its implications for cultural and technological evolution, we must also account for these informal traditions. Here, we have focused on two such practices, body‐based units of measure and heuristics (rules of thumb), by which we hope to emphasize that various non‐standardized solutions to measurement have been available to human societies worldwide. Undoubtedly, this only scratches the surface of a variegated topic, and many other kinds of informal measures, from buckets and baskets to barrels, have been in widespread use.

Shifting the focus from formal to informal measurement systems allows us to understand the origins of measurement as rooted in practical problem‐solving, offering insights into how humans have measured and interpreted their social and ecological environments from prehistoric times to the present. Due to the lack of material evidence, informal units of measure have received relatively little scholarly attention. We argue that the study of everyday measurement practices requires much more focus in cognitive anthropology and archaeology. This area offers ample opportunities for further research. For one, body‐based units used in prehistory may be reconstructed from preserved artifacts like spears, paddles, or bows—contexts where ethnographic studies frequently document body‐based measurements. The widespread use of informal measures also raises an important question: If these units are often sufficient for practical purposes, what exactly has driven the push for standardization? The answers likely lie in the complex interplay of factors such as the need for standardized workflows in mass production, administrative control for governance and taxation, economic demands in trade and construction, scientific classification, and other forces that have shaped the diverse history of measurement, all topics that warrant further research.

## Note

1 One possible exception is commerce, where bottom‐up standards may have emerged as early as the Bronze Age through the use of metal fragment weights (Ialongo & Lago, 2021). However, such “standards” seem to have at best been approximations of roughly defined quanta, mainly used to speed up transactions—a precise standard would be unnecessary when both parties in a transaction own a scale. Later developments in coinage would also have introduced standards to everyday life (Mundell, 2002).
